# Double-in vitro maturation increases the number of vitrified oocytes available for fertility preservation when ovarian stimulation is unfeasible

**DOI:** 10.1038/s41598-020-75699-x

**Published:** 2020-10-29

**Authors:** Nathalie Sermondade, Michaël Grynberg, Marjorie Comtet, Constance Valdelievre, Christophe Sifer, Charlotte Sonigo

**Affiliations:** 1 Department of Cytogenetic and Reproductive Biology, Hôpital Jean Verdier, Hôpitaux Universitaires Paris-Seine-Saint-Denis, Assistance Publique—Hôpitaux de Paris, 93143 Bondy, France; 2 Department of Reproductive Medicine and Fertility Preservation, Hôpital Jean Verdier, Hôpitaux Universitaires Paris-Seine-Saint-Denis, Assistance Publique—Hôpitaux de Paris, 93143 Bondy, France; 3 Department of Reproductive Medicine and Fertility Preservation, Hôpital Antoine Béclère, Hôpitaux Universitaires Paris-Sud, Assistance Publique—Hôpitaux de Paris, 92140 Clamart, France; 4 Université Paris-Sud, Université Paris Saclay, 94276 Le Kremlin Bicêtre, France; 5 Inserm U1133, Université Paris Diderot, 75013 Paris, France; 6 Inserm U1185 Université Paris-Sud, Université Paris Saclay, 94276 Le Kremlin Bicêtre, France; 7 Present Address: Department of Reproductive Biology, Hôpital Tenon, Hôpitaux Universitaires Est Parisien, Assistance Publique—Hôpitaux de Paris, 75020 Paris, France

**Keywords:** Endocrine reproductive disorders, Breast cancer

## Abstract

When ovarian stimulation is unfeasible, in vitro maturation (IVM) represents an alternative option for fertility preservation (FP). This retrospective study aims to evaluate the feasibility of performing within a short time frame two IVM cycles for FP. Seventeen women with breast cancer, 18–40 years of age, having undergone 2 cycles of IVM followed by oocyte vitrification were included. Non parametric analyses were used. No difference was observed between IVM1 and IVM2 outcomes. No complication was reported. The respective contributions of IVM1 and IVM2 for the number of cryopreserved oocytes were comparable irrespective of the delay between both procedures, even when performed during the same menstrual cycle. Those findings suggest that repeating IVM cycles may constitute a safe option for increasing the number of vitrified mature oocytes for FP. These two retrievals may be performed during the same cycle, providing additional argument for a physiologic continuous recruitment during follicular development.

## Introduction

Cancer treatments, such as chemotherapy, may induce early ovarian follicular depletion and impaired reproductive potential of survivors. Fertility preservation (FP) techniques have been developed to improve their possibilities of becoming genetic parents after healing^1,2^. Among them, the most established is cryopreservation of mature oocytes after controlled ovarian stimulation (COS)^1,3^. Although the actual success rate of oocyte vitrification in oncologic situations is unknown, it is commonly admitted that the more oocytes cryopreserved, the better the chances of live birth after thawing. As a consequence, two or more oocyte retrievals are often proposed in order to increase the total number of vitrified oocytes before the initiation of gonadotoxic treatments^4^. However, waiting for a new menstrual cycle before starting a second round of ovarian stimulation is often impossible for oncologic purposes. In this context, random start stimulation has been tried, based on the observation of small antral follicles, possibly responsive to exogenous FSH, anytime during the menstrual cycle^5,6^. Further, the concept of the Duostim emerged, consisting in the repetition of 2 ovarian stimulations within the same menstrual cycle. Initially proposed in infertile poor responder patients^7,8^, the Duostim has been extended to FP^9^. Even if the superiority of this strategy versus two stimulations during two consecutive cycles remains to be demonstrated in infertile patients, it constitutes a method to increase the number of cryopreserved oocytes during the shortest time frame.

In some oncologic situations, COS cannot be considered, as a result of contraindication to exogenous gonadotropins administration or when cancer treatment is urgent. Therefore, alternative options have been proposed, such as oocyte vitrification after unstimulated in vitro maturation (IVM) and/or ovarian tissue cryopreservation^10,11^. Although data about IVM for FP remain scarce, it is likely that the number of cryopreserved oocytes may also be a crucial issue and approaches resulting in an increase in this number could represent a significant achievement.

The present investigation aims to evaluate the feasibility, safety and effectiveness of performing, within a short time frame, two successive unstimulated IVM cycles before chemotherapy, in young women diagnosed with breast cancer.

## Results

### Patients’ characteristics

Overall, mean age of the population was 32.6 ± 5.0 years (23–40 years) (Table 1). Mean serum anti-Müllerian hormone (AMH) levels and antral follicle counts (AFC) performed on the day of oncofertility counselling irrespective of the menstrual phase were 3.0 ± 1.7 ng/mL and 22.8 ± 8.7 follicles, respectively.

**Table 1:** Clinical characteristics of patients that underwent dual IVM for fertility preservation.

Age (years)	32.6 ± 5.0 (23.4–40.0)
BMI (kg/m^2^)	22.2 ± 3 .0 (19.6–29.4)
Antral follicle count	22.8 ± 8.7 (10–38)
Serum anti-Müllerian hormone levels (ng/mL)	3.0 ± 1.7 (0.8–6.3)

Data are expressed as mean ± SD (min–max).

### IVM results

IVM results are reported in Table 2. A similar number of follicles were punctured during both cycles, allowing the retrieval of a similar mean numbers of COCs following IVM1 and IVM2 (7.7 ± 4.5 and 7.8 ± 5.5, respectively *(p* = *0.90)*). After 24 and 48 h of IVM, the mean total numbers of metaphase II oocytes available for vitrification were comparable (4.8 ± 3.2 and 4.4 ± 4.2, p = 0.75). As expected, the total number of vitrified oocytes per patient was significantly higher following two IVM cycles (9.2 ± 6.1 *vs.* 4.8 ± 3.2, *p* < *0.001*). Among the total number of vitrified oocytes, a mean of 57.0% (0–100) and 43.0% (0–100) were obtained following IVM1 and IVM2, respectively.

**Table 2:** Biological outcomes of IVM procedures.

IVM outcomes	IVM1	IVM2	*P*
Number of cumulus oocyte complexes recovered	7.7 ± 4.5	7.8 ± 5.5	*0.9*
Oocyte retrieval rate (%)	36.8 ± 18.3	32.4 ± 16.6	*0.6*
Total number of matured oocytes (MII)	4.8 ± 3.2	4.4 ± 4.2	*0.7*
Total oocyte maturation rate (%)	62.7 ± 23.9	51.6 ± 32.4	*0.4*
Percentage of mature vitrified oocyte per procedure/total number of mature vitrified oocyte	57%	43%	*/*

Data are expressed as mean ± SD.

Oocyte retrieval rate = number of retrieved oocytes / AFC × 100.

Total oocyte maturation rate = number of mature oocytes/number of retrieved oocytes × 100.

Wilcoxon matched-paired signed rank tests were used.

### Timing between two successive oocyte retrievals

**Figure 1:**
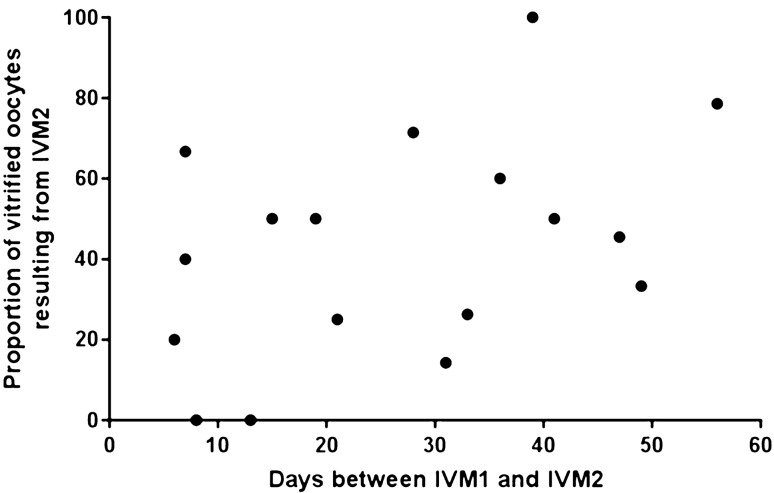
Association between the time between IVM1 and IVM2, and the proportion of vitrified oocytes resulting from IVM2.

Median time between IVM1 and IVM2 was 28.0 days (6.0–56.0). There was no association between the time between both procedures and the proportion of vitrified oocytes resulting from IVM2 (r = 0.39, *p* = *0.11*) (Fig. 1). IVM1 and IVM2 were performed during the same or different menstrual cycles for 6 and 11 patients, respectively. There was no difference between the respective contributions of IVM1 and IVM2 in either situation. The second IVM provided 45.9 ± 29.9% *vs.* 37.7 ± 24.1% of the total number of cryopreserved oocytes, when performed during the same or different menstrual cycles, respectively, *p* = *0.67*.

## Discussion

Although it was commonly thought that antral follicles recruitment occurs in a cyclic manner following the luteo-follicular transition, increasing data challenges this theory. In 2003, Baerwald et al. ultrasonographically documented follicular waves throughout the ovarian cycle^12^, paving the way for other theories. This finding was followed by clinical case reports of successful luteal phase oocyte retrieval for IVM or after COS^13,14^. Based on these data, novel ovarian stimulation regimens have been developed, such as random-start protocols^5,6^ and double stimulation in the same menstrual cycle (Duostim)^7,8^. Used in order to maximize the number of oocytes for poor prognosis patients and/or to facilitate urgent FP procedures, the feasibility of these protocols relies on two further theories of follicle recruitment: (i) the waves theory, according to which many cohorts of antral follicles are recruited during the menstrual cycle or (ii) the continuous recruitment theory, hypothesizing that follicles are recruited continuously throughout the menstrual cycle^15^. The present investigation confirms that recruitable antral follicles are present within the ovaries throughout the menstrual cycle, endowed with oocytes capable of IVM. The delay between both immature retrievals was not associated with the success of the second procedure, even when less than 10 days separated the two procedures, providing additional arguments for a continuous follicular recruitment.

Data from in vitro fertilization (IVF) indicates that the number of oocytes is a key point for success. Cumulative live birth rates following IVF or egg donation continue to increase with the number of oocytes harvested^16–18^, suggesting that the higher the oocyte yield, the better the likelihood of achieving a live birth. However, the generalization of those results obtained with fresh oocytes to frozen eggs may be questioned. Although, data on the outcomes of oocyte vitrification cycles is scarce, evidence indicate that the number of cryopreserved oocytes represents a crucial issue for the success of FP strategies^19^. In this context, it is likely that approaches resulting in an increase in this number could represent a significant achievement. In particular, some authors applied the double stimulation protocol for FP patients, aiming at maximizing the number of vitrified oocytes without delaying cancer treatments^9^. However, few data are currently available regarding safety of COS in neoadjuvant situations, still leading to reluctance for some oncologists to agree with this strategy, especially if a double stimulation is planned within a limited time frame. Other options can then be proposed, including unstimulated IVM or ovarian tissue cryopreservation or a combination of both techniques^20^. Although preliminary, our findings suggest that double IVM might constitute a feasible and safe option for increasing the total number of cryopreserved oocytes, especially in cases where patients are reluctant to undergo a surgical procedure for ovarian tissue cryopreservation.

The present results should be confirmed in a larger population referred for FP. In addition, even if all procedures were only performed by experienced practitioners in our center, the unpredictable nature of immature oocyte retrieval might have influenced the recovery rate between IVM1 and IVM2 and further results of each procedure. Few data are currently available regarding the outcome of cryopreserved oocytes. In particular, the competence of oocytes retrieved during the luteal phase was initially questioned. Recent data indicates that luteal phase ovarian stimulation results in similar euploid blastocyst formation^8,21^ and similar miRNomic signatures within follicular fluids^22^. Although it still merits our attention, reassuring obstetrical and neonatal data have also been preliminary outlined^23,24^. However, the competence of IVM oocytes remains poorly known, even if live births were recently reported^25^.

In conclusion, unstimulated IVM represents an alternative for preserving fertility of breast cancer patients when ovarian stimulation is unfeasible. Our findings suggest that repeating IVM cycles may constitute a feasible and safe option for increasing the total number of cryopreserved mature oocytes, a key point to success. In addition, the second IVM can be performed during the same menstrual cycle, even within a short time frame (less than 10 days), providing additional argument for a continuous follicular recruitment throughout the menstrual cycle. Further analyses should be performed to evaluate the relevance and efficiency of this strategy in terms of live births.

## Methods

### Patients

From July 2013 and July 2017, we studied 17 women, 23 to 40 years of age, having undergone 2 cycles of IVM followed by oocyte vitrification before a first chemotherapy for breast cancer. All of them were characterized by early referral for oncofertility counseling. A first IVM cycle (IVM1) was scheduled rapidly, irrespective of the menstrual cycle phase^26^, while waiting for therapeutic decision. At this time, oncologists did not give agreement for ovarian stimulation. Finally, when COS could not be considered due to urgent chemotherapy and/or oncologic reasons, patients were offered a second IVM cycle (IVM2) combined with ovarian tissue cryopreservation. The present retrospective study was approved by our Local Ethic Committee (CLEA-2017-46), all methods were performed in accordance with the relevant guidelines and regulations and informed consent was obtained from all subjects.

### Ovarian reserve assessment

Measurement of serum AMH and transvaginal ovarian ultrasound scan for AFC were systematically performed, irrespective of the menstrual phase, on the day of oncofertility counseling (before IVM1) and just before IVM2. Serum AMH levels were determined using a fully automated AMH assay (Elecsys AMH; Roche Diagnostics International). The limit of detection, limit of quantification, and maximum imprecision were 0.01 ng/mL, 0.03 ng/mL, and 3.5%, respectively. Ultrasound scans were performed using a 5–9 MHz multi-frequency transvaginal probe (RIC 5–9-D; Voluson E8 Expert, General Electric Medical Systems, Paris, France) by one single operator, who was blinded to the results of hormone assays. The objective of ultrasound examination was to carefully and exhaustively determine the number and sizes of small antral follicles in both ovaries.

### Oocytes in vitro maturation and vitrification procedure

Oocyte retrieval was performed as previously described for all IVM procedures^27^, irrespective of their rank. Briefly, cumulus-oocyte complexes (COCs) were recovered transvaginally under ultrasound guidance. All visible follicles were fully cleared out. After 24 h of incubation in IVM culture medium (Origio, Denmark) enriched with 20% inactivated patient’s serum and FSH and LH (Menopur, Ferring, Germany)^28^, COCs were denuded. Mature metaphase II oocytes were frozen using Kitazato Vitrification Kit (Kitazato BioPharma Co., Ltd., Japan) according to manufacturers’ instructions, and loaded in a minimal volume on the Cryotop device. Vitrification was induced by immediate plunging into liquid nitrogen^29^. Oocytes having failed to mature after 24 h were kept for 24 additional hours of culture. After 48 h, oocytes that have reached metaphase II stage were frozen.

### Statistical analysis

Continuous data are presented as mean ± standard deviation (SD) and categorical data are presented as percentages. Wilcoxon matched-paired signed rank tests were used to compare IVM1 and IVM2 outcomes, as well as the total number of mature vitrified oocytes after one or two IVM cycles. The respective proportion of vitrified oocytes resulting from IVM1 or IVM2 was calculated as the number of metaphase II oocytes following IVM1 or IVM2, divided by the total number of vitrified oocytes after both procedures. The correlation between the delay separating both oocyte retrievals and the gain of a second IVM procedure was assessed by Spearman test. Mann Whitney test was used to compare the gain of IVM2 when performed during the same or the subsequent menstrual cycle. A p-value < 0.05 was considered significant.
